# Novel Receptor-Derived Cyclopeptides to Treat Heart Failure Caused by Anti-β_1_-Adrenoceptor Antibodies in a Human-Analogous Rat Model

**DOI:** 10.1371/journal.pone.0117589

**Published:** 2015-02-20

**Authors:** Valérie Boivin, Niklas Beyersdorf, Dieter Palm, Viacheslav O. Nikolaev, Angela Schlipp, Justus Müller, Doris Schmidt, Vladimir Kocoski, Thomas Kerkau, Thomas Hünig, Georg Ertl, Martin J. Lohse, Roland Jahns

**Affiliations:** 1 Institute of Pharmacology and Toxicology, University of Würzburg, Würzburg, Germany; 2 Rudolf-Virchow-Center/DFG-Research-Center for Experimental Biomedicine, University of Würzburg, Würzburg, Germany; 3 Institute for Virology and Immunobiology, University of Würzburg, Würzburg, Germany; 4 Institute of Pathology, University of Würzburg, Würzburg, Germany; 5 Comprehensive Heart Failure Centre (CHFC), University Hospital of Würzburg, Würzburg, Germany; 6 Lehrstuhl Anatomie I, University of München (LMU), München, Germany; San Diego State University, UNITED STATES

## Abstract

Despite recent therapeutic advances the prognosis of heart failure remains poor. Recent research suggests that heart failure is a heterogeneous syndrome and that many patients have stimulating auto-antibodies directed against the second extracellular loop of the β_1_ adrenergic receptor (β_1_EC2). In a human-analogous rat model such antibodies cause myocyte damage and heart failure. Here we used this model to test a novel antibody-directed strategy aiming to prevent and/or treat antibody-induced cardiomyopathy. To generate heart failure, we immunised n = 76/114 rats with a fusion protein containing the human β_1_EC2 (amino-acids 195–225) every 4 weeks; n = 38/114 rats were control-injected with 0.9% NaCl. Intravenous application of a novel cyclic peptide mimicking β_1_EC2 (β_1_EC2-CP, 1.0 mg/kg every 4 weeks) or administration of the β_1_-blocker bisoprolol (15 mg/kg/day orally) was initiated either 6 weeks (cardiac function still normal, *prevention-study, n = 24 (16 treated vs. 8 untreated)*) or 8.5 months after the 1st immunisation (onset of cardiomyopathy, *therapy-study, n = 52 (40 treated vs. 12 untreated)*); n = 8/52 rats from the therapy-study received β_1_EC2-CP/bisoprolol co-treatment. We found that β_1_EC2-CP prevented and (alone or as add-on drug) treated antibody-induced cardiac damage in the rat, and that its efficacy was superior to mono-treatment with bisoprolol, a standard drug in heart failure. While bisoprolol mono-therapy was able to stop disease-progression, β_1_EC2-CP mono-therapy -or as an add-on to bisoprolol- almost fully reversed antibody-induced cardiac damage. The cyclo¬peptide acted both by scavenging free anti-β_1_EC2-antibodies and by targeting β_1_EC2-specific memory B-cells involved in antibody-production. Our model provides the basis for the clinical translation of a novel double-acting therapeutic strategy that scavenges harmful anti-β_1_EC2-antibodies and also selectively depletes memory B-cells involved in the production of such antibodies. Treatment with immuno-modulating cyclopeptides alone or as an add-on to β_1_-blockade represents a promising new therapeutic option in immune-mediated heart failure.

## Introduction

Heart failure (HF) is a major cause of hospitalization and death; overall 50% of the patients die within four years of diagnosis [1]. HF may result from various causes and pathologies and is therefore considered a heterogeneous syndrome rather than a single disease entity. The presence of auto-antibodies directed against the β_1_-adrenergic receptor (β_1_-AR) apparently identifies a subgroup of patients at risk [2]. The β_1_-AR mediates most of the cardiac effects of the catecholamines adrenaline and noradrenaline, which are often highly elevated and predict unfavorable prognosis in HF [3,4]. Whereas short-term β_1_-AR stimulation improves cardiac performance, its chronic activation leads to progressive deterioration of cardiac structure and function [5].

During the past decade evidence has accumulated that many HF patients have functionally active autoantibodies directed against and stimulating the cardiac β_1_-AR (anti-β_1_-abs) [6,7,8]. Such anti-β_1_-abs are found particularly in patients with idiopathic dilated cardiomyopathy (DCM), which is characterised by dilatation and impaired contraction of the left or both ventricles [9]. The presence of stimulating anti-β_1_-abs is associated with reduced cardiac function [10], ventricular arrhythmias [2], sudden cardiac death [2,11], and increased cardiovascular mortality [2]. This suggests a potential for strategies to counteract such harmful receptor-antibodies.

Stimulating anti-β_1_-abs almost exclusively target the second extracellular loop of the β_1_-AR (β_1_EC2), which is the largest and most structured of the three extracellular receptor loops and, thus, may represent a readily accessible target on the cell surface [12,13]. Moreover, β_1_EC2 contains T- and B-cell epitopes [14,15]. Recent data derived from the receptor’s crystal structure underscore that β_1_EC2 is essential for the stabilisation and locking of the receptor’s agonist binding pocket [13,16]. Thus, it seems conceivable that anti-β_1_EC2 may allosterically induce an active state of the β_1_-AR [12,17]. Immunisation of Lewis rats against the β_1_EC2 gives rise to stimulating anti-β_1_EC2, and within 8 months antibody-positive rats develop progressive cardiac dilatation, wall-thinning, and loss of contractile function typical for DCM [18]. Isogenic transfer of anti-β_1_EC2 to naїve Lewis rats likewise induced HF in recipients [6,18].

To target such harmful antibodies, we conceived a novel peptide-based strategy aiming to specifically neutralise disease-inducing autoantibodies, in particular anti-β_1_EC2. In this aim we generated peptide-homologs of β_1_EC2 and cyclised them to increase their stability *in vivo* [19] and to better mimic the epitope-structure, and then investigated whether they might prevent or have a therapeutic effect (alone or -to better mimic the clinical situation- as add-on to β_1_-blocker therapy) in our rat model of anti-β_1_EC2-induced HF.

## Materials and Methods

### Generation and characterisation of β_1_EC2-cyclopeptides

Linear peptides comprising 24 amino-acids of the human β_1_EC2-sequence (AA199 to 222; ARAESDEARRCYNDPKCCDFVTNR**G**)[20] were synthesised commercially on a Multiple Peptide Synthesizer (SYROII, MultiSynTech GmbH, Witten, Germany) using the solid phase Fmoc protocol with side chain protected Fmoc amino-acid derivatives on Rink Amide MBHA resins (Novabiochem-Merck Biosciences GmbH, Bad Soden, Germany). For cyclisation of the peptide on the solid phase, an additional Fmoc-Glu-ODmab was incorporated at the C-terminal end of the linear peptide; after selective removal of the Dmab side chain, the resin-bound linear peptide was treated with diisopropyl-carbodiimide and N-hydroxy-9-azabenzotriazole in N,N’-dimethyl-formamide for several hours. The cyclisation process was monitored by repeated Kaiser’-tests [59]. Cleavage from the synthesis resin generated a peptide amide; the protective groups of the cyclopeptide were removed by treating the resin with trifluoro-acetic acid/triisopropylsilane/ ethandithiole/water for 2 hours. The generated cyclopeptide β_1_EC2-CP was analysed by high pressure liquid chromatography (HPLC), and by mass spectrometry (MALDI-MS).

A cyclic peptide corresponding to the β_2_EC2-sequence (comprising AA182 to 204; RATHQEAINCYANETCCDFFTNQ**G**)[16] was synthesized and purified along the same lines and served as a control for specificity.

### Study-protocol and generation and characterisation of anti-β_1_-EC2-antibodies

Fusion-proteins (FP) between glutathion-S-transferase (GST) and the 2^nd^ extracellular loop of the human β_1_-AR (β_1_EC2; AA195-225)[20] served as immunisation agent (β_1_EC2/GST-FP). The study-protocol and guideline-conform animal housing conditions were approved by the local authorities (Vote No. 621-2531.01-35/04, Experimental Animal Use and Care Committee, Government of Lower Franconia, Bavaria, Germany).

In brief, n = 76 two months old Lewis/CrlBR rats were either s.c. immunised with 50 μg β_1_EC2/GST-FP, or n = 38 rats were control-injected with 0.9% NaCl (t = 0). To maintain high anti-β_1_EC2-titers, all immunised rats were boosted with β_1_EC2/GST-FP (or 0.9% NaCl) every month over 20 months as previously described [18]. Application of the different linear or cyclic β_1_-AR peptides (corresponding to the primary AA-sequence of either the first (β_1_EC1) or the 2^nd^ extra-cellular β_1_-AR loop (β_1_EC2)) or the β_1_-receptor blocker bisoprolol was initiated either 6 weeks after the 1^st^ immunisation (i.e. 15 days after the 1^st^ boost, *prevention-study*), or 8.5 months after the 1^st^ immunisation (*therapy-study*). At 4 week-intervals the animals received either *preventive* (n = 24, **treatment arms *a, b*, and *f* only**) or *therapeutic* (n = 52, treatment arms *a-f*) interventions over 12 months with **(*a*)** β_1_EC2-CP (1.0 mg/kg intravenously (i.v.)), **(*b*)** bisoprolol (15 mg/kg/day orally, derived from titration pre-experiments [5/10/15mg] as oral dose decreasing heart rate by at least 10%, e.g., 15mg: from 236±10 to 205±9 bpm; n = 5, p<0.005), **(*c*)** β_1_EC2-Lin (1.0 mg/kg i.v.), **(*d*)** β_1_EC1-Lin (1.0 mg/kg i.v), ***(e)*** β_1_EC2-CP (1.0 mg/kg i.v.) **plus** bisoprolol (15 mg/kg/day orally) co-treatment, or **(*f*)** no specific treatment (immunised “positive”controls).

For treatment groups **(*a)***, **(*b)***, and **(*f*)** in total n = 38 non-immunised rats were injected with 0.9% NaCl and treated in parallel (“negative” controls). Blood was taken at regular intervals; rat-IgG was prepared by caprylic acid precipitation and assayed for reactivity by ELISA or competition-ELISA against linear peptides corresponding to the human β_1_EC2-sequence (AA199-223)[12], and by immuno-fluorescence microscopy with human embryonic kidney (HEK)293 cells stably expressing 0.4 pmol β_1_-AR/mg membrane protein (HEKβ_1_-cells) [18]. Specificity of the anti-β_1_EC2-abs was confirmed by co-localisation experiments using previously characterised N-terminal rabbit anti-β_1_-abs [21]. Bound antibodies were detected with appropriate species-specific secondary antibodies (Dianova, Hamburg, Germany; anti-rabbit-, or anti-rat-Fab_2_, conjugated to Cy2 or Cy3). Calibrated rat-IgG (Dianova) served to quantify specific IgG-antibodies.

The effects of anti-β_1_EC2 on β_1_AR-mediated intracellular cAMP-production were assessed by measuring fluorescence resonance energy transfer (FRET) in HEKβ_1_-cells transiently transfected with a FRET-sensor for cAMP, Epac1-camps [8]. The sensor consists of the cAMP-binding protein Epac1 flanked by enhanced cyan or yellow fluorescent protein. 48 h after transfection with Epac1-camps, cAMP measurements were performed microscopically as described [8]. The cells were maintained in FRET-buffer supplemented with 50 nM ICI 118551 (Sigma, Deisenhofen, Germany) to block the small level of endogenous β_2_-AR (∼0.1 pmol/mg membrane protein). IgG-preparations were added to the cells at 0.13 μg/μl protein concentration; 2μM (-)isoproterenol (Sigma) was used as a reference to determine the maximal cAMP-response. To test the blocking-efficacy of β_1_EC2-CP on anti-β_1_EC2-induced adrenergic signaling, the different IgG-preparations were pre-incubated with β_1_EC2-CP (20 μg/μl) for 6 h at 4^°^C; for pharmacological blockade of anti-β_1_EC2-induced signals we utilised 5.0μM bisoprolol.

### Echocardiography and haemodynamic measurements

Echocardiograms were obtained from lightly anaestetised rats (30 mg/kg ketamine-HCl and 5 mg/kg xylazine i.p.) with a Vevo770 system (Visual Sonics Inc., Toronto, Canada) equipped with a 17.5 MHz transducer as previously described [18], always by the same experienced echocardiographer, who was blinded to the treatment groups. In brief, the rats were lightly anaestetised (30 mg/kg ketamine-HCl and 5 mg/kg xylazine i.p.), shaved (chest), and placed supine on a special table. M-mode tracings were recorded at baseline (before immunisation), and subsequently every four months in the parasternal long and short axis views according to the the guidelines of the American Society for Echocardiography [22]. Pulsed-wave Doppler spectra were recorded from the apical five-chamber view and the velocity-time integral (VTI) of the transaortic flow served to calculate cardiac output (CO [ml/min] = Aortic VTI x (π [LV-outflow tract diameter/2]^2^) x bpm). Reproducibility of the echocardiographic measurements was assessed as previously described [18]; intra- and interobserver variabilities were <2% or <5%.

Fourty-eight to 72 h after the final echo-Doppler examinations the rats underwent left heart catheterisation using a 3.5 F high-fidelity catheter (Millar Instruments, Houston, Texas) as described in [18]. LV-pressure tracings were recorded digitally over 15 min and analysed off-line (PowerLab, A.D. Instruments, Castle Hill, Australia) [18].

### Macroanatomy and histology of rat tissues

After further deep anesthesia (70 mg/kg sodium pentobarbital i.p.), the hearts were quickly removed, rinsed with ice-cold relaxing-buffer (5% dextrose, 25 mM KCl in PBS), and weighed. The apex was cut, frozen in isopentane (-56°C), and stored at -80°C for further analysis (binding and gene expression studies); the remainig tissue was fixed in 10% PBS-buffered formalin (24–28 h). After the hearts all other relevant inner organs (e.g., lung, liver, spleen, kidneys, brain, and eyes) were removed, rinsed with ice-cold PBS, weighed, sectioned, and fixed for further histologic analysis.

### Heart morphometry, histology, and TUNEL assay

From paraffin-embedded heart preparations cavity- and wall-dimensions were determined by computer-aided analysis of H&E-stained mid-ventricular 2μm-sections as previously described [18]. H&E-stained paraffin-sections also served to quantify damaged and fibrotic cardiac areas (scars). For detection of mast cells, deparaffinised cardiac sections were stained with acidified (pH<2.5) toluidine blue 0.1% (Sigma). The number of toluidine-positive cells was normalized to square millimeter of cardiac tissue. TUNEL-positive cells were quantified in 2μm mid-ventricular paraffin-sections using a TMR Red in Situ Death Detection Kit (Roche, Basel, Switzerland). Only the total number of TUNEL-postive cells/section was determined without further differentiation of the specific apoptotic cell type (e.g., fibroblasts, endothelial cells, cardiomyocytes).

### Membrane preparation and radioligand binding studies

Membranes from apical cardiac tissues from rats of each study-group were prepared as previously described [18]. To determine total β-AR density (B_max_), 35μg membrane protein were incubated (1.5 h, 25°C) in binding buffer with 200pM of the non-selective β-AR antagonist ^125^I-cyanopindolol (^125^I-CYP; Perkin Elmer Life and Analytical Sciences, Billerica, MA). Non-specific binding was determined with 5μM unlabeled L-propranolol. The proportion of β_1_- and β_2_-AR subtypes was estimated from biphasic competition-curves with 10^-10^ to 10^-2^ M of the unlabeled β_1_-selective antagonist CGP20712A (Sigma). The reaction was stopped by rapid filtration (Whatman GF/C filters) and washing with ice-cold buffer. Filter-bound radioactivity was measured by γ-counting. Estimates of maximal binding (B_max_) and the proportion of β_1_- and β_2_-AR-subtypes were calculated with GraphPad Prism 5.00 (San Diego, CA).

### Cardiac gene expression

Total RNA was isolated from frozen myocardium by RNeasy mini Kit (Qiagen, Hilden, Germany). Reverse transcription of total RNA isolated from frozen myocardium was performed in 96-well plates utilising a high capacity RNA-to-cDNA master mix (Applied Biosystems, Foster City, CA). PCR reactions were conducted in the presence of the fluorescent dye Sybr-Green (Cambrex BioScience, East Rutherford, NY) and the reference-dye 6-carboxy-X-rhodamine (ROX) using an ABI PRISM sequence detection system 7700 (Applied Biosystems). All amplification products were controlled for specificity by running a melting curve analysis; results were calculated using the 2^-ΔΔCT^ method. The relative expression levels were derived from a standard curve and normalised to GAPDH as an endogenous control. Quantitative real-time PCR (qRT-PCR) analyses are presented as fold change compared to untreated (0.9% NaCl-injected) control hearts.

The primer-sequences were as follows (5’−3’): β_1_-AR sense: ATGGGTGTGTTCACGCTCTG, anti-sense: CAGCCAGTTGAAGAAGACGA; GRK2 sense: AGAGGGATGTCAATCGGAGA, anti-sense: AAGACCATCTGCCAGTCCAG; GRK5 sense: ACCCTCCCTTCGTTCCAG, anti-sense: ACTTGGACCATACGGACGAT; IL1-β sense: AAATGCCTCGTGCTGTCTG, anti-sense: TCGTTGCTTGTCTCTCCTTG; TGF-β1 sense AAGAAGTCACCCGCGTGCTA, anti-sense: TGTGTGATGTCTTTGGTTTTGTCA.

### Detection of antigen-specific CD4^+^ T-cells and memory B-cells as well as antibody-secreting plasma cells, and plasmablasts

**(a) Recall-assays with T-cells** from spleens of immunised untreated vs. β_1_EC2-CP-treated animals were conducted as described in [60]. In brief, to purify CD4^+^ T-cells from the splenic cell preparations, B-cells and CD8^+^ T-cells were depleted by commercially available magnetic beads (MACS, Miltenyi Biotec, Bergisch-Gladbach, Germany) yielding a purity >85%. 1x10^5^ of the purified CD4^+^ T-cells were then co-incubated in 96-well plates with 1x10^6^ irradiated thymic antigen presenting cells (prepared from a younger rat). Reagents added in the different assays were 1.0 μg/ml β_1_EC2-CP, 1.0 μg/ml tuberculin purified protein derivative (PPD, internal control), 1.0 μg/ml glutathion-S-transferase (GST), as well as 1.0 and 0.1 μg/ml GST/β_1_EC2-fusion proteins (FP), respectively. Measured T-cell reactivities were normalised to medium. After 48 h of incubation the cells were pulsed with 1.25 μCi/well [^3^H]-thymidine and further incubated for 16 h before the cells were harvested; the DNA-incorporated radioactivity was measured using a beta-plate counter.

**(b) For ELISPOT assays** plates were coated overnight with either 1.8 μg/ml anti-rat IgG (H+L) or the specific antigens (GST/β_1_EC2-FP or GST alone) in 50 mM Tris-buffer, pH 9.4. The plates were then washed 3 times, blocked with BSA (3h, 37°C), and incubated overnight at 37°C with splenocytes or bone marrow cells from rats treated as indicated in RPMI 1640/X-VIVO-15 medium supplemented with 10% FCS (1x10^3^ to 10^6^ cells per well). After 12 h the cells were discarded and the plates were washed several times (PBS/0.5% Tween) before an alkaline phosphatase-conjugated secondary anti-rat-IgG antibody (0.3 μg/ml) was added to detect bound rat IgG. After further incubation (3h, 37°C) and washing-steps (3 times PBS/0.5% Tween) the plates were developed using LMP/BCIP 5:1 (1.0 ml per well; LMP, low melting agarose; BCIP, 5-bromo-4-chloro-3-indolyl phosphate *p*-toluidine salt, a blue-colored dye).

**(c) For flow cytometric detection of β**_**1**_ **EC2-specific memory B-cells**, splenocytes (1.5x10^8^ cells per staining, 2x10^7^ cells/ ml in PBS/0.1% BSA/0.02% NaN_3_) were first labelled with anti-rat IgG(Fc) PE (Jackson Immunoresearch, West Grove, USA) followed by three washings and a blocking step using normal rat serum plus GST (1:500 and 2 μg/ ml, respectively). After another washing step, OX-33 FITC (BD, Heidelberg, Germany, 0.1 μg/ml) and DyLight649-labelled GST/β_1_EC2-FP (0.2 μg/ml) were added. All incubations were carried out for 15 min on ice and in the dark. Finally, the cells were washed three times and analysed on a FACS-Calibur (BD, Heidelberg, Germany) and FlowJo Software (Tree Star, Ashland, USA) was used to analyse the data.

**(d) To detect functional β**_**1**_ **EC2-specific memory B-cells *in vivo***, B-cells were first purified from total splenocytes by negative depletion using mAb V65, R73, 10/78 and WT.5 plus magnetic beads (MACS, Miltenyi Biotec, Bergisch-Gladbach, Germany) yielding average purities of 87%. 2.6x10^7^ purified B cells were injected in 250 μl PBS i.v. into antigen-naïve syngeneic male Lewis rats. To selectively trigger the transferred memory B-cells, recipient rats were immunised after five days with only 12.5 μg GST/β_1_EC2-FP in adjuvans per animal. Serum was collected for antibody detection just before as well as three and seven days after immunisation. Rats without (memory) B-cell transfer did not produce anti-β_1_EC2 IgG within the seven-day observation period (data not shown).

### Statistical analyses

Data are given as mean ±SEM of at least four animals per treatment-group if not otherwise stated. Statistical analyses were performed using GraphPad Prism 5.00 (San Diego, CA). Functional assays (FRET), molecular parameters (qRT-PCR), as well as haemodynamic and morphometric parameters of antibody-positive vs. corresponding control rats or untreated vs. treated animals, respectively, were analysed by Student’s t-test (where applicable) or repeated measurements one-way ANOVA; significance between the different groups was analysed by Dunnett’s post-test.

Kinetics of anti-β_1_EC2-antibodies and/or the different peptides (where applicable) and differences between functional and echocardiographic parameters (long-term follow-up) were analysed by two-way ANOVA followed by Bonferroni post-hoc testing. Values of p<0.05 were considered statistically significant.

## Results

### Generation and neutralisation of stimulating anti-β_1_EC2-antibodies

To generate the anti-β_1_EC2-induced HF model, we immunised 68 male Lewis/CrlBR rats with fusion-proteins (FP) containing glutathion-S-transferase (GST) and the human β_1_EC2 (amino-acids AA195-225)[20] every month in accordance with institutional guidelines as described before [18]. All immunised rats developed high anti-β_1_EC2-titers (IgG-subclass, for all experiments prepared by caprylic acid precipitation), peaking 5–6 months after the 1^st^ immunisation.

**Fig 1:**
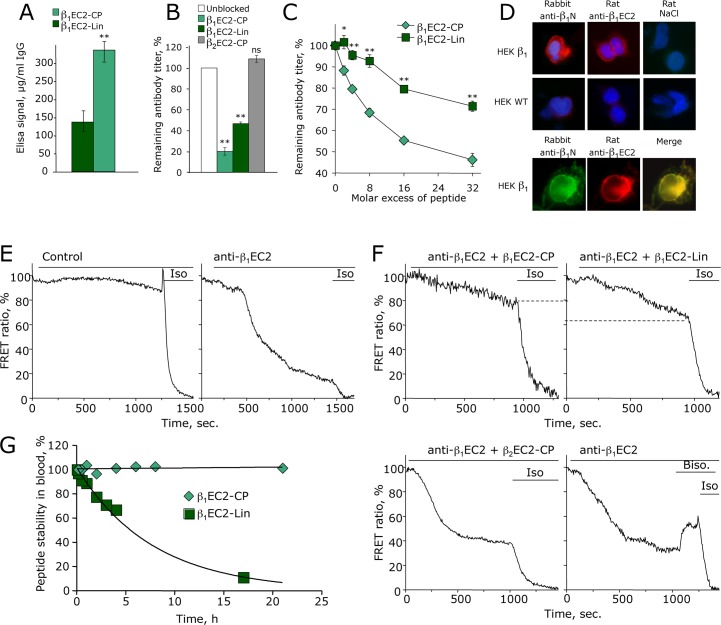
Characterisation of stimulating rat anti-β1EC2. (A) ELISA-reactivity (mean±SEM) of n = 6 representative rat anti- β_1_EC2 (1:5000, 12h, 4°C) with linear (dark green) or cyclic β_1_EC2-peptides (light green); *p*<0.005 (Student’s t-test). (B) Blocking capacity of linear β_1_EC2- or cyclic β_1_- and β_2_EC2 peptide-homologues determined by competition-ELISA with linear β_1_EC2-peptides as antigen (AA199-223); columns are mean titers ±SEM. remaining in the sera after preincubation (12h, 4°C) with a 40-fold excess of β_1_- or β_2_EC2-CP, or β_1_EC2-Lin (n = 20; **p<0.001, one way ANOVA and Dunnett’s post-hoc test). (C) ELISA-competition experiments demonstrating the concentration-dependent blockade of rat anti- β_1_EC2 after preincubation with increasing amounts of linear (squares) or cyclic β_1_EC2-peptides (diamonds); error bars indicate mean ±SEM of n = 3; *p<0.01; **p<0.001 (two way ANOVA and Bonferroni post-hoc test). (D) Immunostaining experiments with rat anti- β_1_EC2 (1:100) using HEK-cells stably expressing β_1_-ARs (HEK β1) or not (wild type; HEK WT). IgG was prepared from anti- β_1_EC2-positive rats or 0.9% NaCl-injected control rats. Amino-terminal β_1_AR-specific rabbit anti-bodies (1:250) served as positive controls [21]. (E) Changes in cAMP levels in HEK β_1_-cells by rat anti- β_1_EC2. HEK β_1_-cells were transfected with the cAMP-sensor Epac1-camps [8], which reacts to cAMP-binding with a reduction in fluorescence resonance energy transfer (FRET) between its fluorophores cyan (CFP) and yellow fluorescent protein (YFP). Anti- β_1_EC2-induced activation of β_1_-AR causes increases in cytoplasmatic cAMP, resulting in a decrease in FRET. Representative FRET-ratio traces of 1/8 experiments with rat anti- β_1_EC2 and control IgG are shown. (F) Blockade of anti-β_1_EC2-induced cAMP-signals after pre-incubation with β_1_EC2-CP, β_1_EC2-Lin, β_2_EC2-CP (12h, 4°C) or 5.0μM bisoprolol. Representative FRET-ratio traces of 1/5 experiments are shown. Values are given in % of maximal cAMP-response achieved with 0.3μM (-)isoproterenol (Iso). (G) Half-life of β_1_EC2-peptides in rat whole blood at room temperature. Rabbit anti-β_1_EC2 served to determine the amount of intact peptides remaining after 2, 10, 30 min, 1, 2, 4, 8, 17, and 22 h by competition-ELISA with linear β_1_EC2-peptides as antigen (duplicate experiments). Half-lifes derived from exponential curve fits were: β_1_EC2-CP, 486 h; β_1_EC2-Lin, 4 h.

Specificity and conformational character of the resultant anti-β_1_EC2 towards β_1_-ARs was ascertained along several lines: by larger ELISA signals with cyclic vs. linear β_1_EC2-peptides (Fig. 1A), by a better recognition of cyclic vs. linear β_1_EC2-peptides in blocking assays (together with a clear preference for β_1_- over β_2_-EC2 sequence (Fig. 1B)), and by a better concentration-dependent neutralization *in vitro* achieved with cyclic vs. linear β_1_EC2-peptides (Fig. 1C). In addition, immunofluorescence-studies confirmed that all rat anti-β_1_EC2 stained native human β_1_-AR in the membrane of stably transfected human embryonic kidney cells (HEKβ_1_-cells), and co-localised with purified β_1_-specific amino-terminal rabbit antibodies [21] (Fig. 1D). Finally, the anti-β_1_EC2 stimulated β_1_-AR-mediated signaling in HEKβ_1_-cells, as evidenced by an increase in cAMP monitored with a co-transfected sensor that shows a decrease in fluorescence resonance energy transfer (FRET) upon binding of cAMP [8]; these signals varied in amplitude and in some cases almost reached the effects induced by the β-AR agonist isoproterenol (Fig. 1E, right panel). No such cAMP-signals were detected with IgG prepared from 0.9%NaCl-injected control rats (Fig. 1E, left panel). Also, control IgG reacted neither with β_1_EC2-peptides in ELISA or competition assays (not shown), nor with β_1_-AR expressed in HEK-cells (Fig. 1D). Stimulation of β_1_-AR/ cAMP signaling by anti-β_1_EC2 was inhibited by pre-incubation with β_1_EC2-peptides, again better by cyclic than by linear peptides (Fig. 1F, top panels); interestingly, this inhibition was more efficient than that achieved by the specific β_1_-AR antagonist bisoprolol (Fig. 1F, bottom right). As internal controls, we also generated cyclic EC2-peptides of the β_2_-AR (β_2_EC2-CP). In ELISA, competition- and FRET-assays, anti-β_1_EC2 was not blocked at all by these β_2_-AR-derived peptides, documenting the β_1_-AR-specificity of the generated antibodies (Fig. 1B and Fig. 1F, bottom left).

Cyclisation of the β_1_EC2-peptides not only improved recognition by the anti-β_1_EC2-anti-bodies but, as predicted from studies on cyclotides from *Viola odorata* [19], also helped to increase their stability in the circulation. Because plasma-half life *in vivo* as determined by ^123^I-(tyrosine)-labeled β_1_EC2 *linear* (t _½_ = 3.9±2.2 min) or *cyclic* β_1_EC2-peptides (t _½_ = 8.1±2.8 min; n.s.) in central venous blood samples (gamma-counted 2, 4, 6, 8, 10, 20, 30, and 60 min after injection of 1.4 to 1.8 MBq ^123^I-labeled peptide/animal, not shown) merely reflected a comparable instantaneous distribution of the respective radiolabeled peptides in the circulation—not considering, e.g., extra-vascular accumulation and/or capillary redistribution (!)—we performed additional *ex vivo* incubation experiments with whole blood to analyse differences in peptide-stability strictly dependent on cyclisation. The latter experiments showed a significantly longer half-life of β_1_EC2-CP (days) than that of its linear counterpart, β_1_EC2-Lin (hours), inferring that cyclopeptides are more resistant to degradation by serum-peptidases than linear peptides (Fig. 1G). Taken together, these data suggested that based on its specificity and longer half-life in blood, the cyclopeptide β_1_EC2-CP might represent a promising drug candidate for our immunisation-induced HF-model.

### β_1_EC2-cyclopeptides prevent and reverse anti-β_1_EC2-induced heart failure

**Fig 2:**
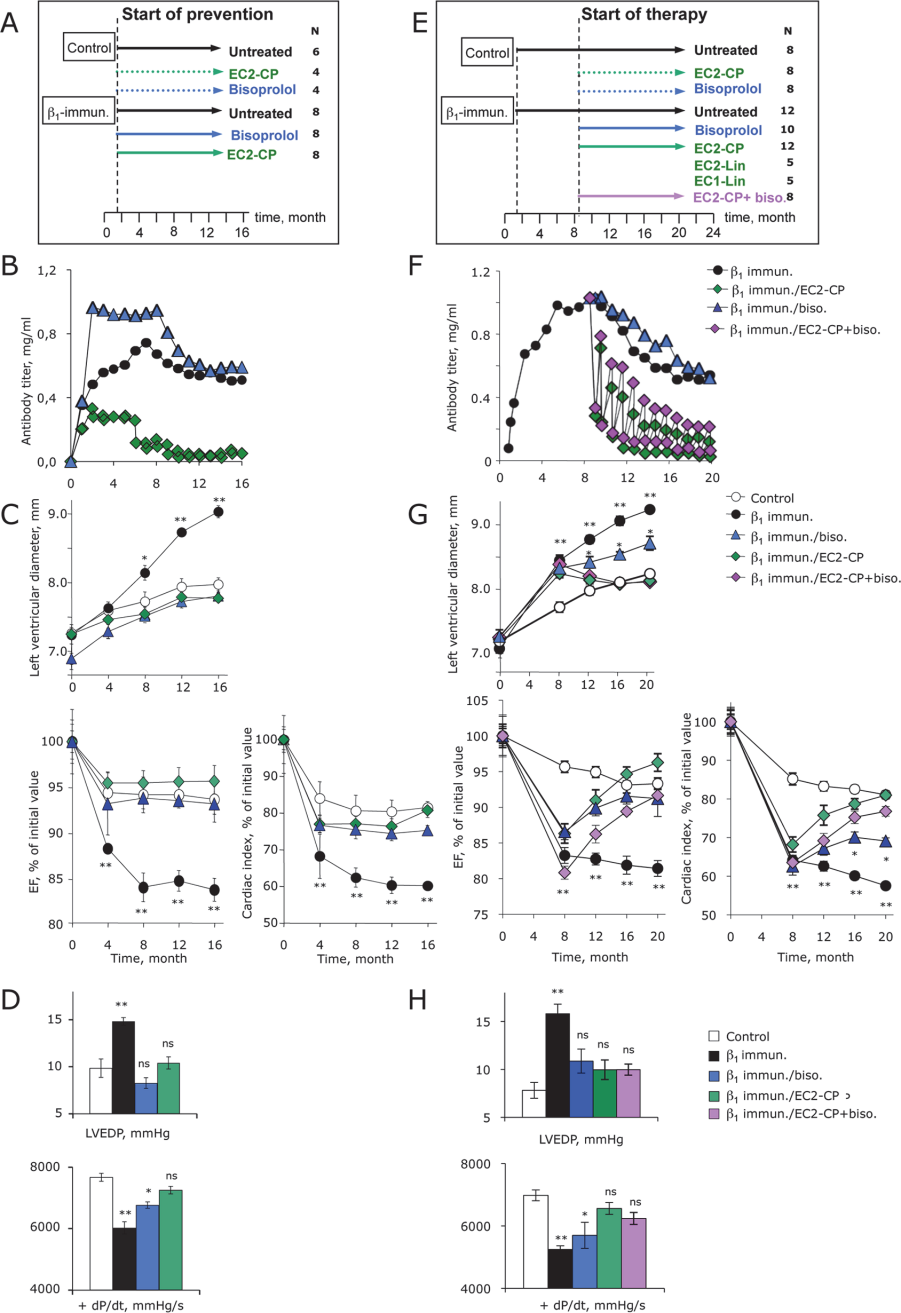
Study protocol and cardioprotection with cyclic β1EC2-peptides. Flow charts of the (A) prevention- and (E) therapy-study with β1EC2-mimicking peptides in the rat. Numbers given refer to animal-numbers in the respective study-groups. Immunisation with β1EC2/GST-FP (or 0.9% NaCl for controls) was started at t = 0. Six weeks or 8.5 months after the 1st immunisation with β1EC2/GST-FP and onset of cardiomyopathy the rats received preventive or therapeutic interventions with the indicated substances every 4 weeks over one year. Immunised untreated animals served as positive, 0.9% NaCl-injected rats as negative controls. Time-course of anti-β1EC2-titers in the (B) prevention- and (F) therapy-study. Titers were measured before and 24h after CP-injection. Values in (B) correspond to the anti-β1EC2-titers in mg/ml over 16 study-months (black circles, untreated; diamonds, β1EC2–CP (1.0 mg/kg/month i.v.); triangles, bisoprolol (15 mg/kg/day orally)). Values in (F) correspond to the anti-β1EC2-titers in mg/ml over 20 study-months (black circles, untreated; green diamonds, β1EC2-CP mono-treated; blue triangles, bisoprolol mono-treated; purple diamonds, β1EC2-CP/bisoprolol co-treated). For better readability error-bars are not shown in the graph. Echocardiography in the (C) prevention- and (G) therapy-study. Graphs show the time-course of the LV end-diastolic diameter (LVED). Lower panels: Time-course of the cardiac index derived from cardiac output/ body weight. Error bars indicate mean ±SEM in the indicated groups; *p<0.01; **p<0.001 (two way ANOVA and Bonferroni post-hoc test). Invasively obtained parameters in the (D) prevention- and (H) therapy-study. Upper panels show LV end-diastolic pressures (mmHg), lower panels contractilility (+dP/dt, mmHg/s). Error bars indicate mean ±SEM of the indicated groups; ns, not significant; *p<0.01; **p<0.001 (one way ANOVA and Dunnett`s post-hoc test).

The protocols of the two studies to either prevent or treat anti-β_1_EC2-induced HF are shown in Fig. 2A and E. The rats were either immunised with β_1_EC2/GST-FP (n = 76) or control-injected with 0.9%NaCl (n = 38), and boosted every month in order to maintain high anti-β_1_EC2-titers. Application of the different peptides or bisoprolol was initiated either 6 weeks after the 1^st^ immunisation (i.e., 15 days after the 1^st^ boost), when cardiac function was still fully normal (called ***prevention-study***, Fig. 2A), or 8.5 months after the 1^st^ immunisation, at the time of cardiomyopathy onset (called ***therapy-study***, Fig. 2E). The peptides (1.0 mg/kg) were injected intravenously every four weeks, bisoprolol (15 mg/kg) was given orally every day (drinking water), and untreated immunised animals received no specific intervention.

Due to the stringent immunisation procedure all rats rapidly developed high titers of anti-β_1_EC2 peaking after 5–6 antigen-boosts, irrespective of bisoprolol-treatment (Fig. 2B and F). In contrast, monthly *preventive* application of β_1_EC2-CPs from their very first injection on abolished a further increase in anti-β_1_EC2-titers, and after 5 injections induced a >80% reduction in free anti-β_1_EC2-abs (vs. initial values, Fig. 2B). After 9–10 β_1_EC2-CP-injections, anti-β_1_EC2-titers had decreased to barely 10% of the initial values despite monthly antigen-boosts (Fig. 2B). In the *therapy-study*, the scavenger effect of β_1_EC2-CP was similar, yielding a progressive decline in anti-β_1_EC2-titers by >70% after two, and by >90% after 4–5 injections (Fig. 2F), irrespective of bisoprolol co-treatment. After each injection, the acute scavenging-effect was visible; in addition, there was a sustained anti- β_1_EC2-decline despite monthly antigen-boosts which resulted in steady state antibody-levels less than 15% of the titers at initiation of therapy. In contrast, therapy with β_1_EC2-Lin produced only negligible effects, and injection of β_1_EC1-Lin (S1 Fig.) or oral mono-treatment with bisoprolol (Fig. 2F, and S2B and S2F Fig.) had no effect on anti- β_1_EC2-titers at all.

In both studies cardiac function was followed every 4 months by echocardiography, and was assessed by left ventricular (LV) catheterisation at month 16 (*prevention-study*) or month 20 (*therapy-study*) as previously described.[18] After 8 months anti-β_1_EC2-positive untreated rats developed LV-dilatation and -dysfunction that progressed continuously in the course of both studies. In the ***prevention-study***, echocardiography and cardiac catheterisation (Fig. 2C and D, and S2 Fig.: A, C, E and G) as well as histomorphology of the hearts (Table 1) of untreated *vs*. treated animals revealed that both bisoprolol and β_1_EC2-CP were able to prevent development of cardiomyopathy and heart failure.

**Table 1:** Macroanatomy and haemodynamic parameters (prevention-study).

	Control	Cont./biso.	Cont./EC2-CP	β1 immun.	β1 immun. /biso.	β1 immun. /EC2-CP
Heart, mg/g	2.55 ±0.03	2.50 ±0.06	2.50 ±0.06	2.78 ±0.04*	2.55 ±0.06	2.46 ±0.05
Spleen. mg/g	1.72 ±0.15	1.79 ±0.05	1.58 ±0.15	1.63 ±0.08	1.74 ±0.05	1.48 ±0.05
Kidney R, mg/g	2.89 ±0.07	2.90 ±0.07	2.79 ±0.01	2.78 ±0.08	2.73 ±0.07	2.79 ±0.06
Kidney L, mg/g	2.74 ±0.10	2.79 ±0.05	2.62 ±0.08	2.68 ±0.14	2.63 ±0.04	2.71 ±0.08
Lung, mg/g	2.86 ±0.09	2.66 ±0.22	2.77 ±0.10	2.89 ±0.12	2.74 ±0.07	2.74 ±0.14
Liver, mg/g	21.18±0.98	22.33 ±0.78	22.51±0.20	24.71±0.98†	22.63±0.46	23.59±0.46
Weight, g.	580 ±31	546 ±8	553 ±11	562 ±4	533 ±27	543 ±24
HF, bpm	246 ±10	173 ±17‡	232 ±7	217 ±4	178 ±9‡	226 ±5
LVSP, mmHg	133 ±6	107 ±11*	135 ±7	119 ±1	106 ±3†	127 ±4
LVEDP, mmHg	9.9 ±1.0	10.6 ±1.5	10.1 ±1.4	14.8 ±0.4‡	8.3 ±0.6	10.4 ±0.6
+dP/dt, mmHg/s	7667 ±130	7012 ±419	7217 ±83	6028 ±23‡	6768 ±106†	7254 ±118
-dP/dt, mmHg/s	6021 ±160	5619 ±155	5986 ±288	5067 ±76‡	5532 ±78*	5887 ±93

The upper part of the table shows the relative (wet) weights in g/kg body weight of selected organs in the groups of the *prevention-study*. Non-immunised 0.9% NaCl-injected rats had no intervention (**Control**) or received treatment with either bisoprolol (**Cont./biso.**) or β_1_EC2-CP (**Cont./EC2-CP**). Similarly, immunised anti-β_1_EC2-positive rats had either no intervention (**β_1_-immun.**) or received bisoprolol (**β_1_-immun./biso.**) or β_1_EC2-CP (**β_1_-immun./EC2-CP**). Values given are mean weights ±SEM of heart, spleen, kidneys (R, right; L, left), lung, and liver, respectively. The lower part of the table shows invasively obtained haemodynamic parameters in the *prevention-study*. Parameters given are (from top to bottom, mean ±SEM): body weight (g), heart frequence (bpm), maximal systolic LV-pressure (mmHg), LV end-diastolic pressure (mmHg), contractilility (+dP/dt, mmHg/s), and relaxation (-dP/dt, -mmHg/s). One way ANOVA and Dunnett`s post-hoc test

Treated vs. control

*p<0.05

†p<0.01

‡p<0.001.

In the ***therapy-study***, β_1_EC2-CP (injected either alone or as add-on to bisoprolol-treatment) almost fully reversed the cardiomyopathic phenotype that had developed before the initiation of therapy, whereas mono-therapy with bisoprolol only stopped further disease-progression. With β_1_EC2-CP (alone or as add-on), echocardiographic LV-dimensions, LV-ejection fraction and cardiac index (Fig. 2G), LV end-diastolic pressure and systolic contraction (Fig. 2H) as well as the heart weight of cardiomyopathic rats returned to control values (Table 2). In contrast, β_1_EC1-Lin or β_1_EC2-Lin failed to elicit any cardiopotective effects (S2 Fig.: B, D, and F). Unlike bisoprolol (alone or as add-on), neither linear peptides nor β_1_EC2-CP decreased heart rate or blood pressure of treated animals (S2 Fig.: G and H).

**Table 2:** Macroanatomy and haemodynamic parameters (therapy-study).

	Control	Cont./biso.	Cont./ EC2-CP	β1 immun.	β1 immun. /biso.	β1 immun. /EC2-CP	β1 immun. /EC2-Lin.	β1 immun. EC1-Lin.	β1 immun. EC2-CP + biso.
Heart, mg/g	2.10 ± 0.04	2.27 ±0.09	2.15 ±0.08	3.18 ±0.22‡	2.93 ±0.11†	2.20 ±0.09	2.88 ±0.24*	2.91 ±0.12*	2.37 ±0.08
Spleen, mg/g	1.31 ± 0.08	1.54 ±0.05	1.18 ±0.03	1.50 ±0.08	1.69 ±0.07	1.23 ±0.04	1.49 ±0.09	1.27 ±0.09	1.62 ±0.07
Kidney R, mg/g	2.34 ± 0.10	2.18 ±0.09	2.22 ±0.08	2.47 ±0.13	2.57 ±0.10	2.25 ±0.05	2.36 ±0.20	2.23 ±0.08	2.37 ±0.07
Kidney L, mg/g	2.28 ± 0.07	2.19 ±0.07	2.19 ±0.08	2.42 ±0.12	2.54 ±0.07	2.29 ±0.06	2.32 ±0.14	2.36 ±0.07	2.27 ±0.06
Lung, mg/g	2.16 ± 0.15	2.07 ±0.06	2.08 ±0.07	2.96 ±0.09‡	2.88 ±0.10*	2.26 ±0.16	2.77 ±0.12*	2.98 ±0.09†	2.35 ±0.08
Liver, mg/g	19.1 ± 0.70	18.5 ±0.55	18.9 ±0.55	22.95 ±0.71†	21.61 ±1.47*	19.83 ±0.50	22.64 ±0.59*	22.89 ±0.41*	20.19 ±0.66
Weight, g	710 ±26	613 ± 22†	695 ±27	648 ±22	598 ±13	673 ±12	598 ±38	689 ±21	606 ±16
HF, bpm	221 ±3	168 ± 6‡	221 ±7	214 ±4	173 ± 3‡	217 ±3	210 ±6	217 ±6	182 ± 2‡
LVSP, mmHg	125 ±6	104 ± 4*	121 ±8	106 ± 3*	108 ± 2*	126 ±4	116 ±4	104 ± 3*	108 ± 3*
LVEDP, mmHg	7.8 ±0.8	9.7 ± 0.9	9.2 ± 1.1	15.8 ± 0.8‡	10.9 ± 1.3	10.0 ± 1.0	14.5 ±1.2†	13.8 ±0.6*	10.0 ± 0.6
+dP/dt, mmHg/s	6979 ±173	6018 ±308	6566 ±416	5249 ± 225‡	5697 ± 231†	6559 ±190	5902 ±171*	5146 ±384‡	6238 ±193
-dP/dt, mmHg/s	5765 ±309	4646 ± 295†	5502 ±248	4079 ± 159‡	4238 ± 117‡	5228 ±166	4857 ±157*	3996 ±106‡	4991 ±106

The upper part of the table shows the relative (wet) weights in g/kg body weight of selected organs in the groups of the *therapy-study*. Non-immunised 0.9% NaCl-injected rats had no intervention (**Control**) or received treatment with either bisoprolol (**Cont./biso.**) or β_1_EC2-CP i.v. (**Cont./EC2-CP**). Similarly, immunised anti-β_1_EC2-positive rats remained either untreated (**β_1_-immun.**) or were treated with (oral) bisoprolol (**β_1_-immun./biso.**) or i.v.-injected with β_1_EC2-CP, β_1_EC2-Lin, or β_1_EC1-Lin (**β_1_-immun./EC2-CP/ EC2-Lin/ EC1-Lin**), or received β_1_EC2-CP/bisoprolol co-treatment (**β_1_-immun./EC2-CP+biso.**). Values given are mean weights ±SEM of heart, spleen, kidneys (R, right; L, left), lung, and liver, respectively. The lower part of the table shows invasively obtained haemodynamic parameters in the *therapy-study*. Parameters given are (from top to bottom, mean ±SEM): body weight (g), heart frequence (bpm), maximal systolic LV-pressure (mmHg), LV end-diastolic pressure (mmHg), contractilility (+dP/dt, mmHg/s), and relaxation (-dP/dt, -mmHg/s). One way ANOVA and Dunnett`s post-hoc test

Treated vs. control

*p<0.05

†p<0.01

‡p<0.001.

**Fig 3:**
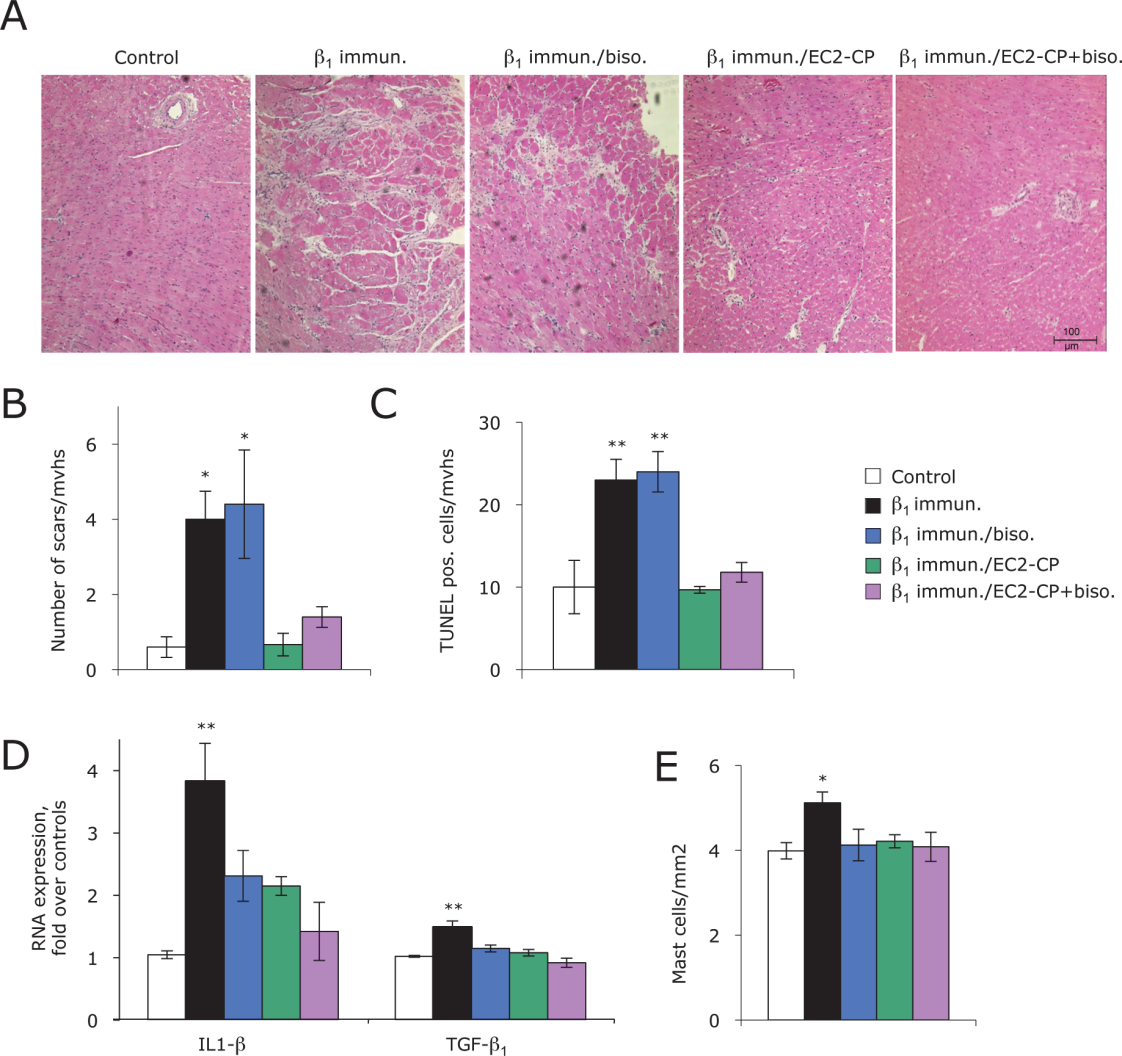
Histology of rat hearts (therapy-study). **(A)** Representative H&E-stained 2μm cross-sections of hearts (from the left to the right) from a control rat, an immunised untreated rat, a bisoprolol- and a β_1_EC2-CP mono-treated rat, and a β_1_EC2-CP/bisoprolol co-treated rat. **(B)** Quantification of fibrotic scars in midventricular 2μm-sections (mvhs, mid-ventricular heart-section). Columns ± error bars represent the mean number of fibrotic scars ±SEM per 3 heart sections in the indicated groups. **(C)** TUNEL-assay with 2μm-mvhs. Columns ± error bars represent mean numbers ±SEM of apoptotic cells (kind of cells not specified) /3 mvhs. **(D)** qRT-PCR of apical cardiac tissues. Columns ± error bars represent the expression of the indicated pro-fibrotic markers ±SEM in the indicated treatment-groups normalised to controls. **(E)** Mast cells in 2μm paraffin-mvhs from the indicated treatment-groups; columns ± error bars represent the mean numbers ±SEM of mast cells/mm^2^ cardiac tissue. For all parameters shown, differences between the treatment-groups were assessed by repeated measures one way ANOVA and Dunnett`s post-hoc test; *p<0.05; **p<0.001.

Morphometry and immunohistology of midventricular 2μm-sections of the hearts analysed at the end of the *therapy-study* underscored the beneficial effects of β_1_EC2-CP (alone or as add-on). The number of myocardial fibrotic scars (Fig. 3A and B) and TUNEL-positive apopotic cells (Fig. 3C) returned to normal levels in β_1_EC2-CP-treated rats. Such a reversal was not seen with bisoprolol mono-therapy (Fig. 3A-C). By contrast, the increases in cardiac transcripts of distinct profibrotic markers (IL1-β, TGF-β1) observed in immunisation-induced HF were reduced by ∼50% with either β_1_EC2-CP or bisoprolol mono-treatment, and even by >70% in the co-treatment group (indicating an synergistic anti-inflammatory effect, Fig. 3D). Also the documented increase in cardiac mast cells in immunized rats was reversed to control levels by both substances (either as mono- or as co-treatment, Fig. 3E). Further morphometric analysis of the hearts revealed enlarged LV-cavities and wall thinning in untreated cardiomyopathic rats. All these features, including the heart weight, were returned to normal in β_1_EC2-CP-treated animals (Table 2 and Table 3). Moreover, the relative wet weight (Table 2), histology (S3 Fig.), and selected laboratory parameters (S4 Fig.) of other organs than the heart revealed an increase in lung and liver weight in untreated anti-β_1_EC2-positive rats (accompanied by a significant increase in GLDH). These signs of congestion were almost reverted in β_1_EC2-CP-treated, but not in bisoprolol mono-treated animals. Importantly, no β_1_EC2-CP-related pathologies were noted in treated vs. control animals. In particular, neither the kidneys nor other inner organs, nor the eyes of β_1_EC2-CP-treated rats had any signs of damage or organ-toxicity attributable to an accumulation or deposition of anti-β_1_EC2/β_1_EC2-CP-complexes (S3 Fig.).

**Table 3:** Morphometric analysis of rat hearts (therapy-study).

	Control	Cont./biso.	Cont./ EC2-CP	β1 immun.	β1 immun. /biso.	β1 immun. /EC2-CP	β1 immun. /EC2-Lin.	β1 immun. EC1-Lin.	β1 immun. EC2-CP + biso.
LVA, mm2	59.9 ±1.8	58.4 ±0.7	57.4 ±0.7	68.3 ±0.8*	61.9 ±5.2	65.2 ±2.5	71.2 ±3.5†	70.9 ±1.4*	55.4 ±1.7
LVCA, mm2	24.1 ±0.9	22.6 ±0.2	21.3 ±0.1	31.1 ±0.3‡	26.8 ±1.0	25.6 ±1.0	32.7 ±1.3‡	32.4 ±1.5‡	22.5 ±1.5
LVWA, mm2	36.9 ±1.3	35.8 ±0.5	36.1 ±0.7	37.2 ±0.7	35.0 ±4.5	39.6 ±1.7	38.5 ±2.4	38.5 ±0.8	34.8 ±1.6
LVCA / LVA %	39.6 ±0.8	38.7 ±0.5	37.2 ±0.5	45.6 ±0.6‡	43,9 ±0.8†	39.4 ±0.8	46.1 ±0.9‡	45.6 ±1.4‡	37.1 ±1.2
IVS, mm	1.7 ±0.1	1.8 ±0.1	1.7 ±0.1	1.5 ±0.1*	1.7 ±0.1	1.7 ±0.1	1.5 ±0.1*	1.4 ±0.1*	1.6 ±0.2
PW, mm	1.8 ±0.1	1.8 ±0.1	1.9 ±0.1	1.6 ±0.1*	1.5 ±0.1	1.8 ±0.1	1.6 ±0.1*	1.6 ±0.1*	1.7 ±0.1*

Morphometric data of rat hearts harvested at the end of the *therapy-study* were obtained by computer-aided analysis of H&E-stained mid-ventricular 2 μm cross-sections as previously described in detail [18]. Parameters given are (from top to bottom, mean ±SEM): LVA_tot_, total LV-area; LVCA, LV-cavity area; LVWA, LV wall area (including LVCA/LVWA-ratio in %); IVS, thickness of the inter-ventricular septum; PW, posterior wall thickness (one way ANOVA and Dunnett`s post-hoc test

Treated vs. control

*p<0.05

†p<0.01

‡p<0.001.

**Fig 4:**
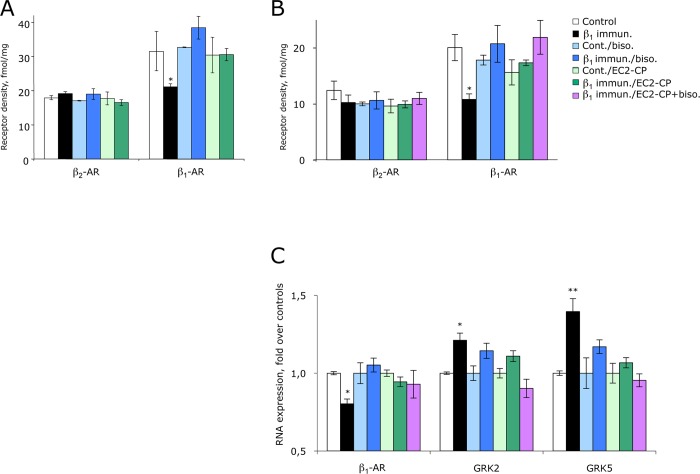
Cardiac adrenergic system and molecular markers. Columns represent the relative amount of β_1_/β_2_-AR subtypes (fmol/mg protein) in cardiac membranes of anti-β_1_EC2-positive immunised rats (A) *preventively* or (B) *therapeutically* treated with either bisoprolol or β_1_EC2-CP alone, or with a combination of both, and corresponding control animals (prevention-arm, month 16; therapy-arm, month 20). Error bars indicate mean values ±SEM of the indicated treatment groups; *p<0.05. (C) Molecular analysis of hearts (apical segments) from treated anti-β_1_EC2-positive rats and corresponding control animals in the therapy-study. Columns ± error bars represent the level of expression of the indicated markers ±SEM in cardiac tissues from the indicated treatment-groups compared to values from 0.9% NaCl-injected rats (controls, set at 100%), as determined by qRT-PCR normalised to GAPDH (one way ANOVA and Dunnett`s post-hoc test; *p<0.05; **p<0.001).

Cardiac failure is usually accompanied by downregulation of cardiac β_1_- but not β_2_-ARs, and by upregulation of cardiac GRKs [4,23,24]. Radioligand-binding studies with ^125^I-cyano-pindolol and selective antagonists showed that β_1_-specific downregulation of β-ARs also occurred in our immunisation-induced HF-model [18], and that this downregulation was largely prevented by (mono-)application of β_1_EC2-CP alone (Fig. 4A and B). As typical for a β-AR antagonist, bisoprolol (mono-)treatment even induced a small increase in cardiac β_1_-ARs, an effect that was fully preserved with β_1_EC2-CP/bisoprolol co-treatment (Fig. 4B). No changes were seen for β_2_-AR under any of the study conditions. Corresponding to the downregulation of cardiac β_1_-AR protein, β_1_-AR mRNA-levels were also significantly reduced in untreated cardiomyopathic rats and returned to normal levels with either β_1_EC2-CP or bisoprolol treatment alone or with β_1_EC2-CP/bisoprolol combination-therapy (Fig. 4C). Moreover, qPCR-analysis of the expression of G protein-coupled receptor kinases (GRKs) involved in counter-balancing sympathetic activity [4,24,25] revealed an upregulation of GRK2 and GRK5 in immunisation-induced HF, which was reverted by bothβ_1_EC2-CP and (to a somewhat lesser extent) by bisoprolol mono-therapy (Fig. 4C), whereas co-treatment with both substances had a clear synergistic effect, resulting even in a slight (non-significant) down-regulation of both GRK’s (Fig. 4C).

### Immunomodulating effects of β_1_-EC2-cyclopeptides

**Fig 5:**
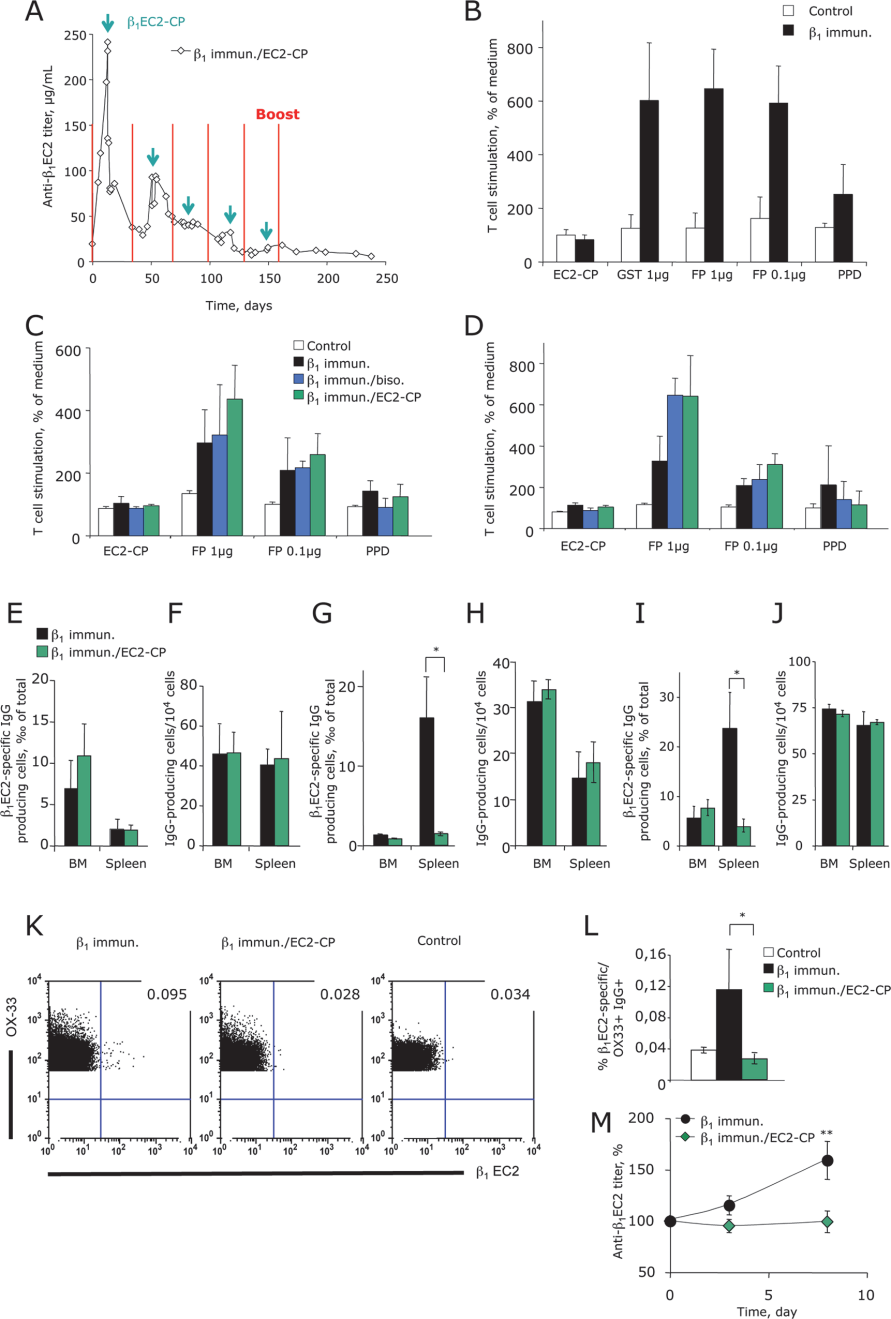
Immunology of cyclic β1EC2-peptides: fate of β1EC2-specific T- and B-cells. **(A)** Titer-course of anti-β_1_EC2-abs in β_1_EC2-CP-treated rats during the first 6 months of the prevention-study *before* and 24h *after* cyclopeptide-injection. Time-points of antigen-boosts are indicated by (red) lines, time-points of CP-injections by (green) arrows. For better readability error-bars are not shown in the graph. **(B)** Pre-experiments (antigenic recall-assays) performed with CD4^+^ T-cells prepared from spleens of GST/β_1_EC2 fusion-protein (FP)-immunised untreated (n = 3, black) vs. 0.9%NaCl-injected control rats (n = 3, white). (**C** and **D**) Reactivities of CD4^+^ T-cells prepared from **(C)** *preventively* or **(D)** *therapeutically* β_1_EC2-CP- (green) or bisoprolol-treated animals (blue) compared to cells prepared from immunised untreated (black) or corresponding 0.9%NaCl-injected control rats (white). Columns ± error bars represent mean T-cell reactivities ±SEM normalized to medium (abbreviations: EC2-CP, cyclopeptide mimicking β_1_EC2; GST, glutathion-S-transferase; PPD, purified protein derivative; FP, GST/β_1_EC2 fusion-protein). (**E** and **F**) ELISPOT-assays to detect long-lived plasma cells (PC) in bone marrow (BM) and spleens of untreated (black, n = 4) vs. β_1_EC2-CP-treated immunised rats (green, n = 4, month 16). Columns in **(E)** depict the fraction of anti-β_1_EC2-secreting PC 14 days after antigen-boost (in ‰ of IgG-producing cells), in **(F)** the total amount of IgG-producing cells per 10^4^cells. Error bars represent mean ±SEM in the indicated groups. (**G** to **J**) ELISPOT-assays carried out with bone marrow cells (BM) and splenocytes prepared from immunised untreated (black) vs. β_1_EC2-CP-treated rats (green) from the prevention- (G and H, n = 2 vs. 3 rats) and the therapy-study (I and J, n = 3 vs. 3 rats). Columns in **(G)** and **(I)** depict the fraction of anti-β_1_EC2-secreting cells 3 days after antigen-boost (vs. IgG-producing cells); columns in **(H)** and **(J)** show the total amount of IgG-producing cells per 10^4^cells. **(K** and **L)** Direct flow cytometric detection of β_1_EC2-specific B-cells. The numbers in the dot plots indicate percentages per quadrant. **(M)** Concentration of anti-β_1_EC2-IgG in the sera of recipient rats after adoptive transfer of B-cells from donors treated as indicated followed by suboptimal immunisation with FP in adjuvans. Error bars represent mean ±SEM in the indicated groups (Student’s t-test, *p<0.05).

To assess whether preventive and/or therapeutic treatment of anti-β_1_EC2-positive rats with the different peptides interfered with the immunopathology of antibody-induced HF, we determined the kinetics of anti-β_1_EC2-abs before and 24 h after peptide-application by ELISA (measuring only free anti-β_1_EC2-abs). In immunised untreated animals, anti-β_1_EC2-titers continuously increased after each antigen-boost (Fig. 2B and F). In line with an antibody-scavenging effect, treatment with β_1_EC2-CP already after two injections significantly decreased, and after 5 injections almost fully suppressed a rise in free anti-β_1_EC2, while bisoprolol treatment did not affect the amount of free anti-β_1_EC2-abs. With preventive and also with (repeated) therapeutic application of β_1_EC2-CP, anti-β_1_EC2-levels remained low despite continued boosting with β_1_EC2/GST-FPs (Fig. 2B and F). This unexpected titer-course, monitored more closely in the first 6 months of the prevention-study (Fig. 5A), suggested that beside its action as a simple scavenger, β_1_EC2-CP also negatively impacted on B- and/or T-cells as the lymphocyte populations responsible for anti-β_1_EC2-production. Moreover, as even preventive injection was initiated after the priming-immunisation and production of class-switched anti-β_1_EC2 of the IgG isotype, β_1_EC2-CP-treatment putatively interfered with the function not of naïve, but of already differentiated memory B-cells, plasma cells (PC), and/or T-cells.

For T-cells, treatment with antigenic peptides has been shown to either induce dominant unresponsiveness of the CD4^+^ T cell-compartment via induction of CD4^+^CD25^+^ regulatory T-cells (T_reg_ cells)[26] or to functionally impair non-T_reg_ CD4^+^ T helper cells [27]. We, therefore, tested the *in vitro* recall responses of purified CD4^+^ T-cells isolated from the spleens of preventively (Fig. 5B and C) or therapeutically (Fig. 5D) treated and untreated rats to the β_1_EC2/GST-FP and its components. CD4^+^ T-cells from both groups clearly responded to the FP but not to β_1_EC2-CP, indicating that the relevant CD4^+^ T-cell epitopes were contained within GST (Fig. 5B-D). Further *in vitro* testing did also not provide any evidence for an induction of β_1_EC2-specific T_reg_ cells by β_1_EC2-CP *in vivo* (not shown).

As the T cell-compartment appeared not to be targeted by β_1_EC2-CP we further analysed the cells directly involved in antibody-production, i.e. B-cells and PC. ELISPOT assays using splenocytes or bone marrow cells freshly isolated from rats two weeks after the last antigen-boost allowed us to determine the frequencies of (mostly) long-lived PC in these organs; however, these experiments revealed that β_1_EC2-CP-treatment did not reduce the frequencies of anti-β_1_EC2-producing PC (Fig. 5E and F).

While long-lived PC express very little or no immunoglobulins on the cell surface [28], B-cells do and could thus serve as direct targets of β_1_EC2-CPs. To detect the few antigen-specific memory B-cells within splenocytes of treated *vs*. untreated rats we differentiated memory B-cells into short-lived plasmablasts by boosting the rats with β_1_EC2/GST-FPs three days prior to the analysis [29]. This allowed us to detect them together with long-lived PC by ELISPOT.

Preventive (Fig. 5G and H) as well as therapeutic (Fig. 5I and J) application of β_1_EC2-CPs resulted in a ∼80% reduction in the frequencies of splenocytes secreting anti-β_1_EC2-abs, which was not achieved with β_1_EC2-Lin (S5A Fig.). As long-lived PC were not targeted by β_1_EC2-CPs (Fig. 5E), this means that more than 80% of the β_1_EC2-specific memory B-cells were affected by this kind of treatment impairing B-cell receptor (BCR)-mediated β_1_EC2-specific memory B-cell expansion and differentiation into anti-β_1_EC2-producing PC. Direct detection of β_1_EC2-specific memory B-cells by flow cytometry (Fig. 5K and L) further revealed complete depletion of this cell population by β_1_EC2-CP treatment. Accordingly, transfer of purified B-cells from immunised β_1_EC2-treated rats into antigen-naïve recipients followed by suboptimal immunisation with β_1_EC2/GST-FP (12.5μg instead of 50μg in adjuvans) did not induce detectable amounts of anti-β_1_EC2-abs in the serum of the recipient rats while B-cells from immunised untreated rats did (Fig. 5M). In summary, our results suggest that β_1_EC2-CP protects rats from immunisation-induced HF, both, by specifically scavenging free anti-β_1_EC2 and by depleting β_1_EC2-specific memory B-cells. It should be noted, however, that the overall numbers of IgG-producing cells were not at all affected by β_1_EC2-CP-treatment (Fig. 5F, H, and J), precluding a general immuno-suppressant effect of the cyclic peptide.

## Discussion

### Autoantibody-induced diseases and current treatment approaches

Autoantibodies directed against self-antigens are the hallmark of many autoimmune diseases, and some of them may even directly cause the disease [30,31]. In Graves‘disease [32], myasthenia gravis [33], and a sub-entity of insulin-resistant diabetes [30] functional autoantibodies (abs) directed against membrane receptors have been recognised as main pathogenetic factors. This illustrates that receptors, proteins that are generally considered as not very immunogenic, can serve as targets for disease-causing abs. This appears to be the case also in a significant number of HF patients, where anti-β_1_AR-abs are suspected to contribute to the development of DCM [2,6,7,8,10,11,18,34,35,36].

Current treatment approaches in autoantibody-induced diseases comprise either targeting the abs themselves, as is the case for immunoadsorption [36], or the cells producing the abs, i.e. short-lived plasmablasts and/or long-lived plasma cells. While plasmablasts and their precursors, i.e. memory B-cells, may be deleted by cytostatic drugs like cyclophosphamide [37] or anti-CD20^+^-abs [38], ablation of long-lived plasma cells requires the use of proteasome-inhibitors [39]. All these therapies present specific problems in terms of unwanted effects and outcome; in particular, proteasome-inhibitors are quite cardiotoxic, rendering them not suitable for the treatment of anti-β_1_AR-mediated HF [40].

Here we present a novel approach to address such disease-causing abs based on cyclic peptides mimicking the relevant target-epitope. We and others have previously shown that immunisation against the 2^nd^ extracellular loop of the β_1_-AR gives rise to receptor-stimulating anti-β_1_EC2 in various animal models [12,14,18,35,41]. Such antibodies appear to allosterically activate the receptors and their signaling cascade, and this activation occurs both in the absence and in the presence of catecholamines [6,12,18]. In the long run, such anti-β_1_EC2 cause myocardial tissue injury, characterised by myocyte apoptosis and fibrosis, myocyte dysfunction, cardiac dilatation, and in the end congestive HF [5,6]. The most likely explanation is that stimulating anti-β_1_EC2 abs cause partial, but chronic activation of cardiomyocyte β_1_-ARs, and thus potentiate the vicious circle of sympathetic activation and HF-progression [3,4,6].

### **Mode of action of** β_1_-**receptor mimicking cyclic peptides**

In our study, monthly injections of β_1_EC2-CP either prevented (*preventive application*) or even reversed (*therapeutic application*) the detrimental consequences of stimulating anti-β_1_EC2. These injections were tolerated well by both immunised anti-β_1_EC2-positive rats and antibody-naïve control animals, and over one year of treatment did not elicit any apparent β_1_EC2-CP-related adverse effects, neither in routine blood laboratory tests nor in a series of organs (S3 and S4 Figs.). In addition, the protection achieved with monthly β_1_EC2-CP-injections was clearly superior to daily applications of 15 mg/kg bisoprolol alone, although mono-treatment with bisoprolol was able to stop the progression of immunisation-induced heart failure. Unlike bisoprolol, β_1_EC2-CP mono-treatment affected neither heart rate nor blood pressure. The beneficial effects of β_1_EC2-CP must at least in part be due to scavenging of anti-β_1_EC2, as is evident from the specific and high-affinity recognition of these antibodies which reflects both the primary sequence of the epitope (by comparison with the β_1_EC1- or β_2_EC2-peptides) as well as the structure (by comparison with the linear β_1_EC2-peptide). In addition, β_1_EC2-CP had a long half-life in blood, which contributes to its scavenging-efficacy.

In addition to its anti-β_1_EC2-neutralizing action, the most intriguing effect of β_1_EC2-CP was the ability to essentially block further anti-β_1_EC2 antibody-production despite continuous antigen-boosts. As detailed in the results-section this failure to respond to the antigen boosts is due to depletion or functional inactivation of β_1_EC2-specific memory B-cells. On a molecular level this depletion is explained by monomeric, e.g. non-productive, stimulation of the BCR [42] precluding an expansion of anti-β_1_EC2-expressing memory B-cells in β_1_EC2-CP -treated animals.

As in the case of direct scavenging, the effect on anti-β_1_EC2-expressing memory B-cells was sequence- and epitope-specific, and was, in particular, not elicited by the corresponding linear peptide (S2 and S5 Figs.).

While direct targeting of PC in anti-β_1_EC2-mediated HF remains a challenge, the cyclo-peptide-approach presented here neutralises the products of short-lived plasmablasts as well as long-lived PC (i.e. existing anti-β_1_EC2-antibodies) and hits β_1_EC2-specific memory B-cells. Although antibody-neutralisation appears sufficient to mediate protection in this model, we hypothesise that depletion of the β_1_EC2-specific memory B-cells may be important for maintaining low antibody-titers and, therefore, enhances the long-term therapeutic efficacy of our approach. By this double action β_1_EC2-CPs not only prevent antibody-induced β_1_-AR activation, but also address the site of antibody-generation, and thereby prevent or revert anti-β_1_EC2-induced cardiac damage. These positive effects were seen in terms of cardiac morphology and function as well as different microscopic, laboratory, and molecular parameters. Moreover, in β_1_EC2-CP-treated animals, together with the restauration of cardiac membrane β_1_-AR, also the increases in GRK2- and GRK5-expression were almost fully reverted. These effects were even more pronounced, when injecting β_1_EC2-CP (as an add-on) to bisoprolol-treatment, resulting in a slight (non-significant) downregulation of both GRK’s. This is of particular interest, as increases in cardiac GRK-transcripts are thought to represent an early adaptation to adrenergic stress preceding β_1_-AR desensitisation [4,43,44]. In addition, increases in cardiac GRK-transcripts have been shown to correlate well with disease severity in HF patients [44], suggesting that β_1_EC2-CP treatment alone or—on a molecular level even more efficient—combined with a β_1_-receptor blocking agent (which corresponds better to the current clinical requirements and treatment guidelines) might also act beneficially in anti-β_1_EC2-mediated human heart failure.

### Chronic heart failure, relevance of the immune system, and new therapeutic strategies

Various factors may contribute to non-ischaemic DCM, including persistent viral infection [45] and autoimmune-mediated damage to myocytes [46,47,48]. These factors may coincide, because autoimmune-reactions to myocardial proteins may be virus-triggered [31,47]. In the last decade a growing number of heart-directed abs and alterations of the immune system have been described in heterogeneous subsets of patients with DCM [34,46,49], indicating that multiple mechanisms may play a role in the pathogenesis of autoimmune-mediated heart failure (HF). Amongst others, abnormalities have been found in cytokines [46], T lymphocyte subsets, and in cells mediating myocardial inflammation [50]. Moreover, in >30% of DCM patients abnormal immune-reactions against distinct cardiac self-antigens have been described, including autoantibodies against myocyte contractile proteins [47,51], mitochondrial proteins [52], and membrane receptors [10,53,54]. However, only a few of them have been shown to cause, in fact, myocardial tissue injury and congestive HF by themselves [18,35,51]. In humans the individual genetic predisposition also significantly influences the susceptibility to self-directed immune-reactions [34,49,55], but the so far available clinical data underscore the pathophysiological and clinical importance of stimulating anti-β_1_AR-abs in HF and the need for novel antibody-directed therapeutic strategies [6,36,56]. We demonstrate here that anti-β_1_EC2-mediated cardiostimulatory effects *in vivo* cannot be efficiently neutralised with β_1_-receptor blockers alone. This finding fits perfectly with *in vitro* data using anti-β_1_EC2 isolated from DCM-patients [8]. Other experimental antibody-directed strategies consist in their removal from the blood by immunoadsorption [36,57,58] or, more recently, in the development of anti-β_1_AR-neutralising small oligonucleotides currently assayed in *vitro* [56].

As a simple alternative, we present here a novel specific and very efficient strategy to treat autoimmune-mediated HF *in vivo* with cyclopeptides mimicking the target-domain of stimulating anti-β_1_EC2-antibodies (administered alone or as an add-on to β_1_-receptor blocker treatment). β_1_EC2-CPs acted as antibody-scavengers in the circulation, but also depleted or functionally inhibited anti-β_1_EC2-expressing memory B-cells involved in antibody-production. Our data suggest that β_1_EC2-CPs (alone or combined with a β_1_-receptor blocking drug) might evolve into a novel save and efficient strategy to neutralise stimulating anti-β_1_EC2 also in human HF. Furthermore, it will be interesting to investigate whether such cyclic peptides might prove to be beneficial also in other autoantibody-mediated diseases.

## Conclusions

By taking advantage of a *human-analogous* rat model the here presented *in vivo*-experiments provide the basis for the clinical translation of a novel double-acting therapeutic strategy for immune-mediated heart failure. Cyclic peptides mimicking the target epitope of functionally active antibodies stimulating the cardiac β_1_-adrenergic receptor on the one hand act as direct antibody-scavengers in the circulation thereby precluding antibody-induced harm from the heart; in addition, the cyclic peptides were found to have a long-term effect by selectively depleting memory B-cells involved in the production of cardio-noxious receptor antibodies. Besides the prevention and/or treatment of immune heart failure (either as a mono-substance or combined with a β_1_-receptor blocking agent) the here presented approach might be helpful also in other autoantibody-mediated diseases.

## Supporting Information

S1 Fig Effect of different peptides on anti-β_1_EC2-antibody titres (therapy-study). Time course of anti-β_1_EC2-titers in the therapy study measured *before* and 24h *after* CP-injections. Absolute antibody-concentrations over 20 study-months are shown (mg/ml; black circles, untreated; green diamonds: β_1_EC2-CP (1.0 mg/kg/month i.v.); blue triangles: bisoprolol (15 mg/kg/day orally); grey squares: β_1_EC2-Lin (1.0 mg/kg/month i.v.); green squares: β_1_EC1-Lin (1.0 mg/kg/month i.v.)); for better readability error-bars are not shown in the graph.
(TIF)

S2 Fig Effect of different peptides on various echocardiographic and haemodynamic parameters in the prevention- and the therapy-study. Echocardiographic follow-up in the **(A,C)** prevention- and **(B,D)** therapy-study. Graphs (A) and (B) show the time-course of LV end-diastolic and end-systolic diameters (LVED, LVES), graphs (C) and (D) the cardiac index (derived from cardiac output/body weight) in the prevention- and the therapy-arm of the study, respectively. Error bars indicate mean ±SEM; *p<0.01, **p<0.001, **p<0.0001 (two way ANOVA and Bonferroni post-hoc test). Invasively obtained haemodynamic parameters in the **(E)** prevention- and **(F)** therapy-study. Panels (from top to bottom) show heart frequence (bpm), maximal systolic LV-pressure (mmHg), LV end-diastolic pressure (mmHg), LV-contractility (+dP/dt, mmHg/s), and -relaxation (-dP/dt, -mmHg/s). Error bars indicate mean ±SEM; *p<0.01; **p<0.001, ***p<0.0001 (one way ANOVA and Dunnett`s post-hoc test).
(TIF)

S3 Fig Histologic analysis of various organs (therapy-study, mono-treatment). Representative H&E-stained 2μm cross-sections from various organs analyzed for treatment-related pathologies. Panels (from top to bottom) show organs analyzed from immunised anti-β_1_EC2-positive untreated, β_1_EC2-CP-treated, or bisoprolol-treated rats. Representative sections from brain, liver, heart, kidney, eye, and spleen after 12 months of treatment are demonstrated. Neither treatment strategy caused detectable organ-specific toxicity or therapy-related pathologies.
(TIF)

S4 Fig Routine laboratory parameters (therapy-study, mono-treatment). Columns ± error bars represent the mean values ±SEM for different laboratory serum parameters in the indicated treatment groups (from left to right, top row: AST, aspartate-aminotransferase; ALT, alanine-aminotransferase; aP, alkaline phosphatase; Crea, creatinine; Urea, urea. Bottom row: GGT, gamma-glutamyltransferase; GLDH, glutamat lactate dehydrogenase; Bili, bilirubin; Myo, myoglobin; CK, creatinine kinase); *p<0.05 (one way ANOVA and Dunnett`s post-hoc test).
(TIF)

S5 Fig Effect of cyclic *versus* linear peptides on splenocytes. ELISPOT-assays carried out with bone marrow cells (BM) and splenocytes prepared from immunised untreated (black, n = 3) vs. β_1_EC2-CP-treated (dark green, n = 5) vs. β_1_EC2-Lin-treated animals (light green, n = 3). Columns in (A) depict the fraction of anti-β_1_EC2-secreting cells 3 days after antigen-boost (in ‰ of IgG-producing cells); columns in (B) show the total amount of IgG-producing cells per 10^4^cells. Error bars indicate mean ±SEM; *p<0.05 (one way ANOVA and Dunnett`s post-hoc test).
(TIF)
