# Supplementary figures and images for: Novel Receptor-Derived Cyclopeptides to Treat Heart Failure Caused by Anti-β1-Adrenoceptor Antibodies in a Human-Analogous Rat Model
Source: PLoS One. 2015 Feb 20;10(2):e0117589. doi: 10.1371/journal.pone.0117589 (PMC4336331; doi:10.1371/journal.pone.0117589)

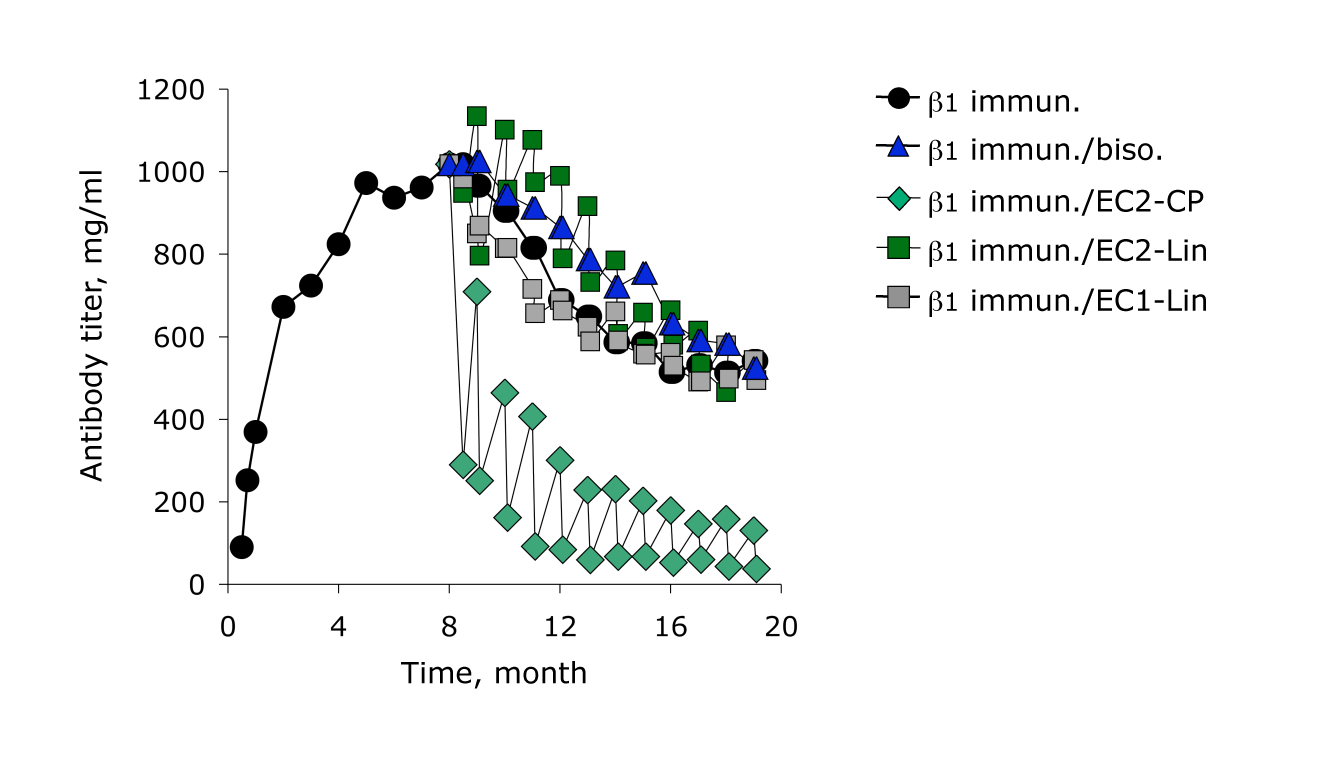

Supplement: S1 Fig — Time course of anti-β1EC2-titers in the therapy study measured before and 24h after CP-injections. Absolute antibody-concentrations over 20 study-months are shown (mg/ml; black circles, untreated; green diamonds: β1EC2-CP (1.0 mg/kg/month i.v.); blue triangles: bisoprolol (15 mg/kg/day orally); grey squares: β1EC2-Lin (1.0 mg/kg/month i.v.); green squares: β1EC1-Lin (1.0 mg/kg/month i.v.)); for better readability error-bars are not shown in the graph. (TIF) [file pone.0117589.s001.tif]

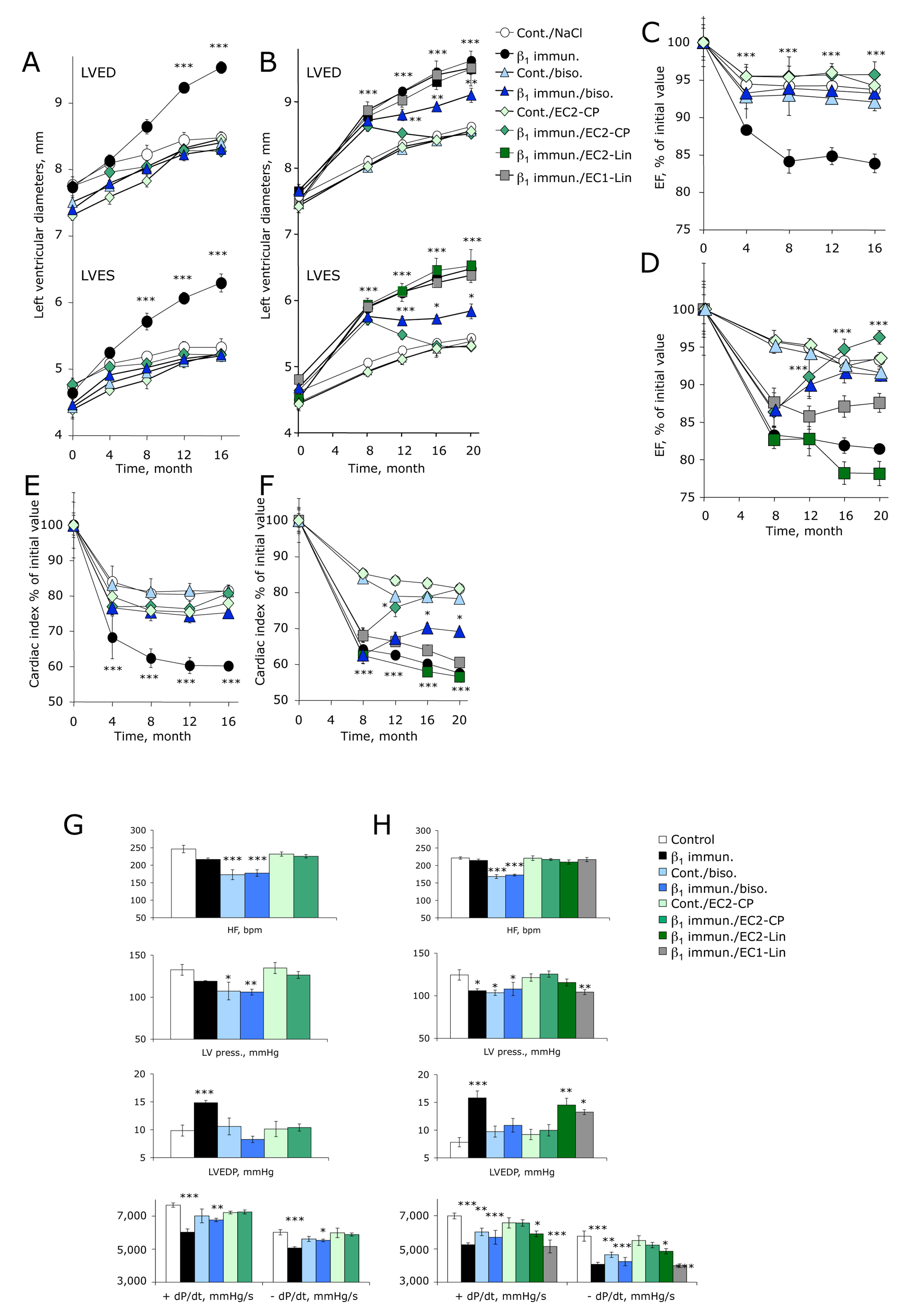

Supplement: S2 Fig — Echocardiographic follow-up in the (A,C) prevention- and (B,D) therapy-study. Graphs (A) and (B) show the time-course of LV end-diastolic and end-systolic diameters (LVED, LVES), graphs (C) and (D) the cardiac index (derived from cardiac output/body weight) in the prevention- and the therapy-arm of the study, respectively. Error bars indicate mean ±SEM; *p<0.01, **p<0.001, **p<0.0001 (two way ANOVA and Bonferroni post-hoc test). Invasively obtained haemodynamic parameters in the (E) prevention- and (F) therapy-study. Panels (from top to bottom) show heart frequence (bpm), maximal systolic LV-pressure (mmHg), LV end-diastolic pressure (mmHg), LV-contractility (+dP/dt, mmHg/s), and -relaxation (-dP/dt, -mmHg/s). Error bars indicate mean ±SEM; *p<0.01; **p<0.001, ***p<0.0001 (one way ANOVA and Dunnett`s post-hoc test). (TIF) [file pone.0117589.s002.tif]

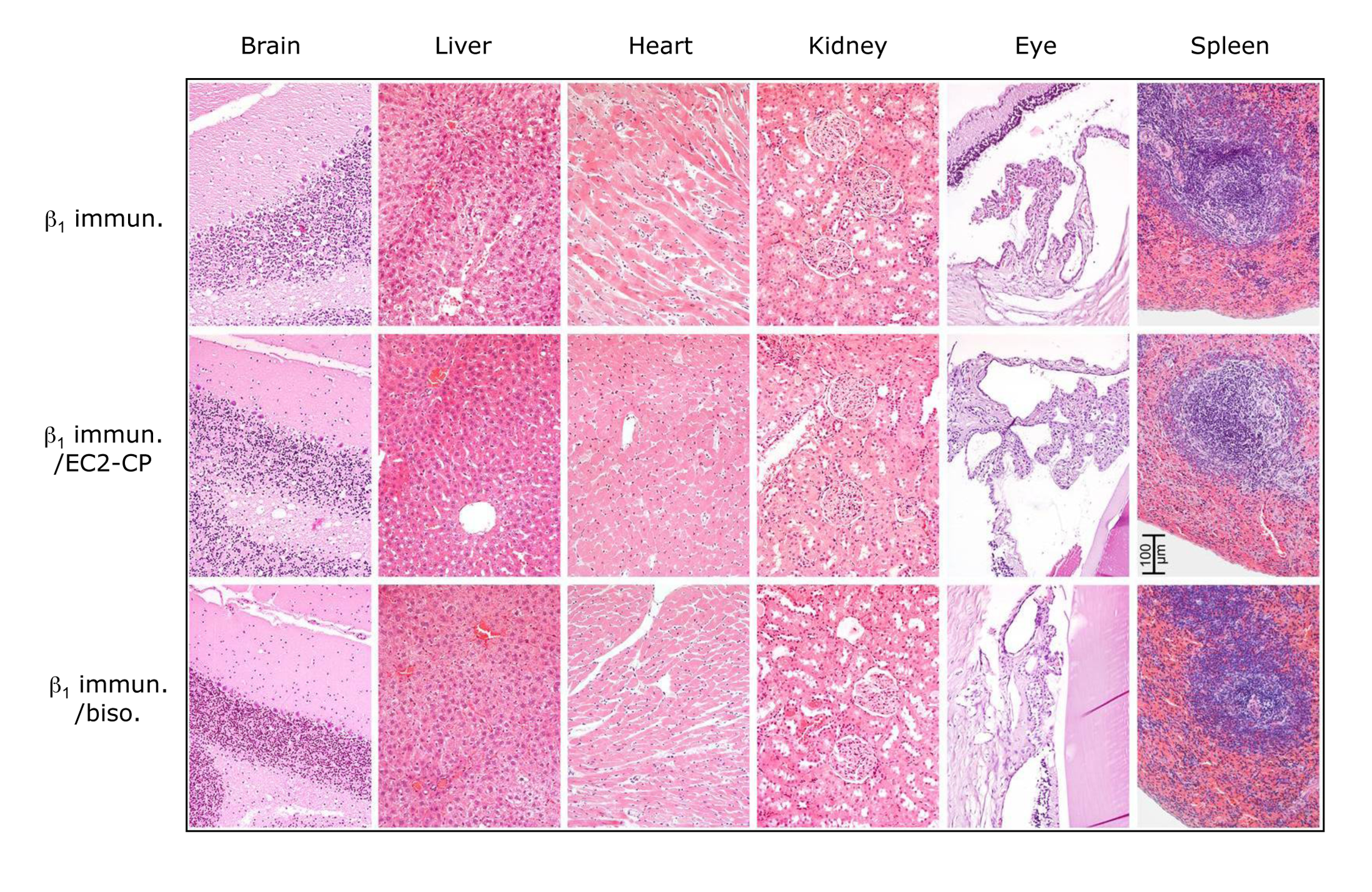

Supplement: S3 Fig — Representative H&E-stained 2μm cross-sections from various organs analyzed for treatment-related pathologies. Panels (from top to bottom) show organs analyzed from immunised anti-β1EC2-positive untreated, β1EC2-CP-treated, or bisoprolol-treated rats. Representative sections from brain, liver, heart, kidney, eye, and spleen after 12 months of treatment are demonstrated. Neither treatment strategy caused detectable organ-specific toxicity or therapy-related pathologies. (TIF) [file pone.0117589.s003.tif]

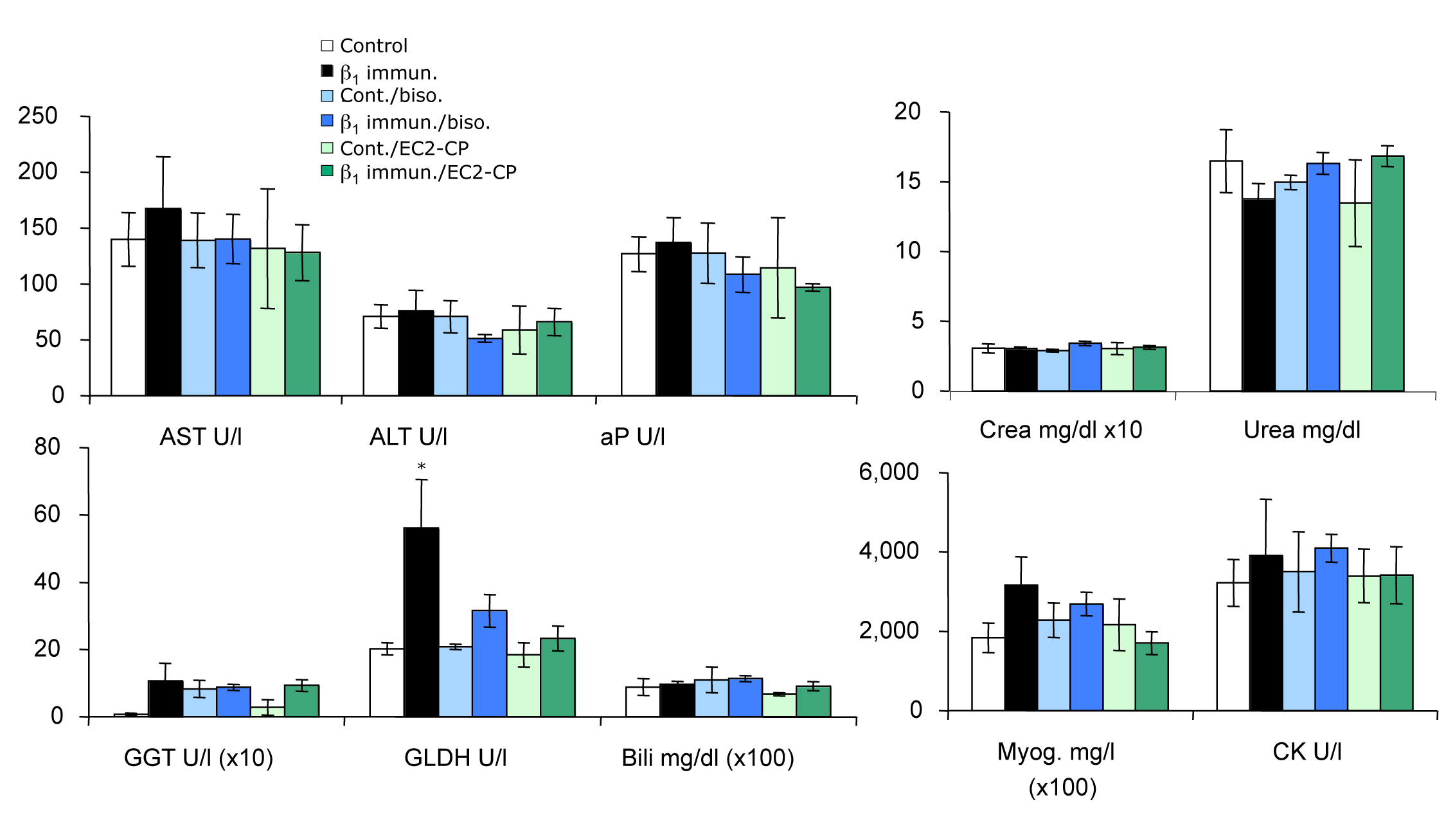

Supplement: S4 Fig — Columns ± error bars represent the mean values ±SEM for different laboratory serum parameters in the indicated treatment groups (from left to right, top row: AST, aspartate-aminotransferase; ALT, alanine-aminotransferase; aP, alkaline phosphatase; Crea, creatinine; Urea, urea. Bottom row: GGT, gamma-glutamyltransferase; GLDH, glutamat lactate dehydrogenase; Bili, bilirubin; Myo, myoglobin; CK, creatinine kinase); *p<0.05 (one way ANOVA and Dunnett`s post-hoc test). (TIF) [file pone.0117589.s004.tif]

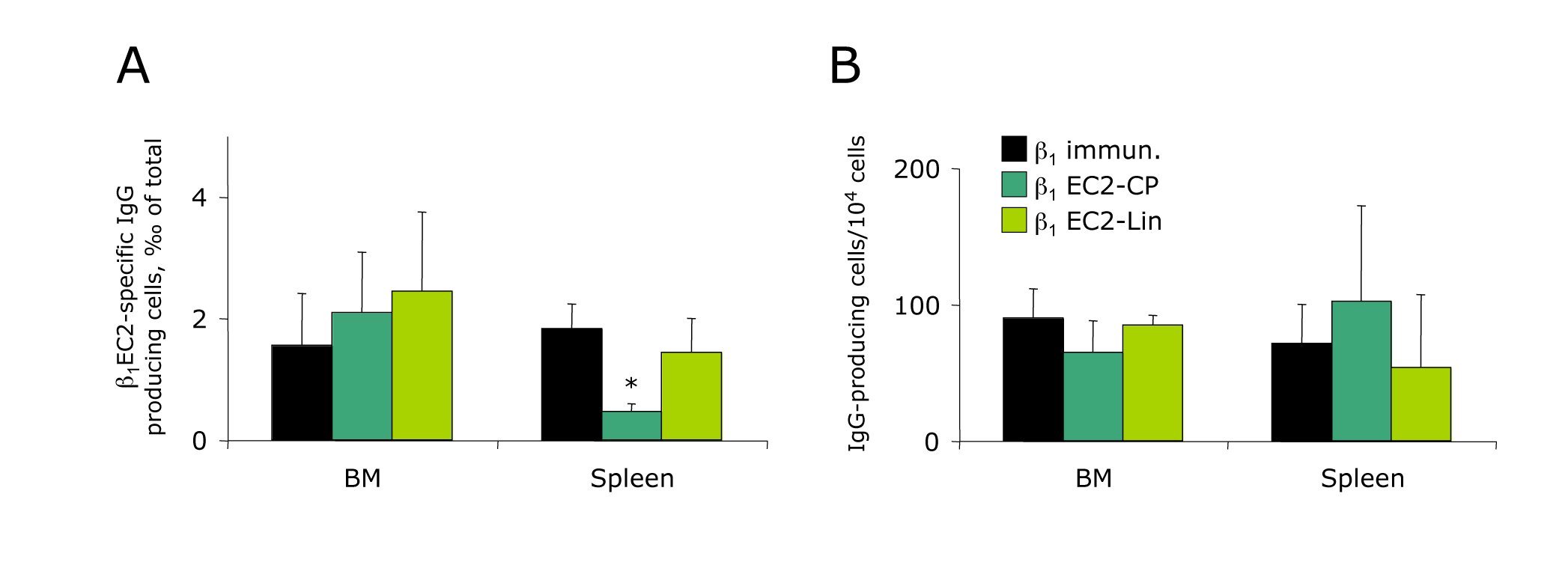

Supplement: S5 Fig — ELISPOT-assays carried out with bone marrow cells (BM) and splenocytes prepared from immunised untreated (black, n = 3) vs. β1EC2-CP-treated (dark green, n = 5) vs. β1EC2-Lin-treated animals (light green, n = 3). Columns in (A) depict the fraction of anti-β1EC2-secreting cells 3 days after antigen-boost (in ‰ of IgG-producing cells); columns in (B) show the total amount of IgG-producing cells per 104cells. Error bars indicate mean ±SEM; *p<0.05 (one way ANOVA and Dunnett`s post-hoc test). (TIF) [file pone.0117589.s005.tif]
